# Supplementary material for: Biomechanics of the Human Osteochondral Unit: A Systematic Review
Source: Materials (Basel). 2024 Apr 8;17(7):1698. doi: 10.3390/ma17071698 (PMC11012636; doi:10.3390/ma17071698)
Supplement: Supplementary file 1 [file materials-17-01698-s001.zip › materials-2822942_Glossary.pdf]

Glossary

Biochemistry: the application of chemistry to the study of biological processes at the cellular and molecular level.

Biphasic: see **Constitutive models**.

Bone Surface on Total Volume (BS/TV): see **microCT**.

Bone Volume on Total Volume (BV/TV): see **microCT**.

Compression: mechanical testing in which a material/tissue is loaded by a plate with a size equal or greater than the one of the sample-of-interest.

Constitutive Models: mathematical frameworks describing the mechanical behavior of a material/tissue in an arbitrary scenario, i.e., mechanical and/or thermal loading conditions. More in detail, constitutive models explain the peculiar trend of the stress-strain relations, therefore providing governing equations, conservation laws and kinematic relations specific of the material mechanical behaviour. Depending on the modeling complexity, these models are defined by multiple parameters – which, in case of biological tissues, are related to the structure and composition – describing specific mechanical characteristics. Models are linear when the relation proposed to describe the stress-strain trend is proportional. Elastic models describe the behaviour of materials/tissues in which the stress depends only on the deformation state. Elasto-plastic models are implemented as extension of the previous ones, to describe materials which do not fully recover the strain after the ending of the stimuli; therefore, these kind of models split the deformation into an elastic and plastic part. The mechanical behaviour of materials/tissues displaying a time-dependence of stress and strain are described by viscous and viscoelastic models. Materials/tissues or tissues showing an irreversible deformation after loading are described by purely plastic constitutive models. The previous models can be combined in order to describe the mechanical response of more complex materials and tissues, i.e., viscoelastic, elasto-plastic, visco-elasto-plastic. Biphasic models describe the stress-strain relation of materials/tissues composed by a mixture of a porous permeable solid matrix and an interstitial fluid. Therefore, and besides the solid matrix contribution, the fluid motion and the relative dissipative factor must be taken into account to describe the comprehensive response of the material/tissue. Poroelastic models are biphasic models assuming an elastic response of the material solid matrix. Fibril-reinforced, non-homogeneous, poroelastic models represent a further refinement of the previous, in which the response of the solid matrix is described by taking into account its specific structure and inner arrangement. In addition to the previous classifications, models can be isotropic, transversely isotropic, or anisotropic, depending on whether their mechanical response to a stimulus is independent, transversely dependent – i.e., along a specific plane of symmetry – or fully dependent on the direction of the stimulus itself.

Delayed Gadolinium Enhanced MRI (dGEMRIC): see **MRI**.

Digital Image Correlation (DIC): full-field optical technique computing the distribution of displacements and, consequently, of strains on the outer surface of a structure, i.e., tissues or (bio)materials. The technique employs image registration algorithms and tracking methods to detect 2D changes induced by a mechanical testing on the structure-of-interest.

Digital Volume Correlation (DVC): innovative full-field technique that requires 3D images of a structure in the undeformed and deformed state, and exploits the images texture or grey levels gradients to compute the inner displacement and strain fields. Despite DVC can be applied to datasets acquired by various imaging techniques, its use is mainly related to X-ray based imaging.

Dual-Energy X-ray Absorptiometry (DEXA/DXA): medical imaging approach using very low levels of X-rays to measure the density of mineralized tissues, i.e., bone. DEXA scan results can provide helpful details about risk for osteoporosis (bone loss) and fractures (bone breaks).

Elastic behavior/properties: see **Constitutive models**.

Ex vivo: experiments or measurements performed on tissue in an artificial environment – i.e., outside the organism – taking care to replicate – as close as the specific investigation allows – the *in vivo* conditions, e.g., temperature, stimuli.

Fourier Transform Infrared (FT-IR): absorption technique that use mid-infrared light to pass through a sample. By the losses of energy due to the wavelengths absorbed by specific molecular bonds, FT-IR can detect the components of a sample.

Glycosaminoglycan (GAG)/Proteoglycan (PG): GAGs are large complex carbohydrate molecules that interact with a wide variety of proteins involved in physiological and pathological processes. GAGs are found on all animal cell surfaces in the extracellular matrix. PGs are composed of a core protein to which one or more GAG chains are covalently attached. Loss of GAG chains of PGs is a phenomena peculiar of early OA.

Histology: the study of tissues and cells under a microscope.

In vitro: experiments or assessments performed by using components of an organism after isolating them from the normal biological environment. Such a procedure allows to investigate more deeply the behaviour of specific units, i.e., cells.

In vivo**:** experimentation performed on living organism in its normal intact state. Animal testing and clinical trials are two forms of *in vivo* research.

Indentation: mechanical testing in which a tissue/material is compressed by an indenter – spherical or plane-ended – with a diameter lower than the size of the sample. Therefore, the contact area between the indenter and the sample is very small.

Magnetic Resonance Imaging (MRI): a medical diagnostic tool using radio waves and a magnet to retrieve detailed pictures of the body inner areas, thanks to specifically designed sequences. MRI is the gold standard technique to investigate soft tissues, e.g., the brain and spinal cord, the heart and blood vessels, joints ligaments and articular surfaces, the organs in the pelvis and abdomen, and the breast. By focusing on joints and articular tissues, MRI sequences like T2 mapping and T1rho allow to retrieve signals – computed as relaxation times – detecting biochemical changes in the articular cartilage before abnormalities are visualized by standard radiographs and morphological MRI. T2 is influenced mainly by free water molecules and, moreover, by the integrity of collagen content, i.e., structure of the collagen network. T1rho is strictly related to the tissue fixed charge density which reflects the PGs content; therefore, through such a sequence water and PGs content – and, moreover, their depletion – are detectable. A contrast agent, such as gadolinium, may be injected to enhance the contrast of specific tissues and organs, therefore requiring specific sequences to image such tissues, e.g., delayed gadolinium enhanced MRI, dGEMRIC.

Mechanical behavior: see **Constitutive models**.

Mechanical parameters: see **Constitutive models**.

Mechanical testing: an experiment used to investigate the mechanical properties of a material/tissue .

Micrometric Computerized Tomography (microCT): an X-ray (radiography) system produces two-dimensional shadow images of complete internal three-dimensional structures, but in a single two-dimensional shadow projection the depth information is completely mixed. Only an X-ray tomography system allows the visualization – and, therefore, the measurement – of three-dimensional structures without requiring the preparation or chemical fixation of the sample. Typically, the spatial resolution of conventional medical Computerized Tomography (CT) scanners falls within the range (1 ÷ 2.5) mm, corresponding to (1 ÷ 10) cubic mm voxel (volume element) size. MicroCT definitely improve such a spatial resolution by seven to ten orders in the volume terms (microns). As in the “macro” CT-scanners, the inner structure of the material/tissue of interest can be evaluated fully non-destructively. By focusing on bone and – more broadly – to the related tissue engineering porous materials and constructs, the main structural parameters that can be retrieved by microCT investigation are the following: Bone Volume (BV) respect to Total Volume (TV) in which it is contained (BV/TV, also known as Bone Volume Fraction, BVF); Bone Surface (BS) respect to Total Volume (TV) in which it is contained (BS/TV); mean Thickness (Th) of Bone Trabeculae (Tb) (Tb.Th); mean Separation (Sp) between Trabeculae (Tb) (Tb.Sp); mean Trabecular Number for distance unit (Tb.N); Structure Model Index (SMI), indicating the relative presence of rods and plates in the trabecular bone structure.

Osteoarthritis (OA): is a degenerative joint disease, in which the articular tissues progressively loss their original features – i.e., structure and composition – and,, therefore, their mechanical response. It is the most common type of arthritis.

Osteochondral (OC) unit: multi-layer structure of the epiphysis of bones articulated in joints. Its main layers (i.e., tissues), listed from the articular surface down, are: articular cartilage (AC), subchondral bone (SB) and trabecular bone (TB).

Quantitative Computed Tomography (QCT): medical technique employing X-ray Computed Tomography (CT). As output of the 3D imaging analysis, QCT provides a measure of Bone Mineral Density (BMD), i.e., by using a specific calibration standard.

Peripheral Quantitative Computed Tomography (pQCT): quantitative computed tomography (QCT) used to estimate the Bone Mineral Density (BMD) in a peripheral part of the body, e.g., ankle, wrist.

Preferred Reporting Items for Systematic Reviews and Meta-Analyses (PRISMA): evidence-based minimum set of features that should be reported in order to perform systematic reviews or meta-analyses. Despite PRISMA is mainly used to evaluate the effects of interventions, it can also be used to summarize the evidence retrieved by systematic reviews with informative purposes. In addition, PRISMA statement uses a flow diagram to outline the selection of the studies through the multiple phases of a systematic review, i.e., number of the records retrieved, included, and excluded.

Raman Spectroscopy: it is an analytical technique using a single wavelength laser to irradiate the sample. By measuring the scattered light generated through the excitation of the molecule bonds it is possible to identify the components of a sample, primarily thanks to their underlying chemistry.

Structure Model Index (SMI): see **microCT**.

T1rho: a quantitative MRI sequence and measurement, see **MRI**.

T2: a quantitative MRI sequence and measurement, see **MRI**.

Tissue homeostasis: a normal and uninflamed state of a tissue, maintained through a self-regulating mechanism that balances the interactions between biological processes and physio-chemical conditions. The onset of pathologies, in particular of degenerative ones, impairs the physiological conditions peculiar of the tissue homeostasis.

Trabecular Number (Tb.N): see **microCT**.

Trabecular Separation (Tb.Sp): see **microCT**.

Trabecular Thickness (Tb.Th): see **microCT**.
