# Supplementary material for: Biomechanics of the Human Osteochondral Unit: A Systematic Review
Source: Materials (Basel). 2024 Apr 8;17(7):1698. doi: 10.3390/ma17071698 (PMC11012636; doi:10.3390/ma17071698)
Supplement: Supplementary file 1 [file materials-17-01698-s001.zip › materials-2822942_List_abbreviation.pdf]

List of Abbreviations

aBV/TV	Axial Bone Volume Fraction
AC	Articular Cartilage
appBVF	Apparent Bone Volume Fraction
BMI	Body-Mass Index
BS/TV	Bone Surface/Total Volume
BV/TV	Bone Volume Fraction
BWM	Best-Worst Method
C	Compression
CC	Confined Compression
D	Dynamic (constitutive model)
DIC	Digital Image Correlation
DVC	Digital Volume Correlation
DXA	Dual-Energy X-ray Absorption (also known as DEXA)
E	Elastic or Young Modulus
E_{dyn}	Dynamic Modulus
E_f	Initial or Strain-dependent Fibril Network Modulus
E_m	Initial or Strain-dependent Non-Fibrillar Matrix Modulus
E_q	Equilibrium Modulus
E_0	Instantaneous Modulus or Strain-dependent Instantaneous Modulus
ECM	Extra-Cellular Matrix
EP	Elasto Plastic (constitutive model)
FRPVE	Fibril-Reinforced Poro-Visco-Elastic (constitutive model)
GAG	Glycosaminoglycan
G'	Shear storage Modulus,
G"	Loss Modulus
H_A	Aggregate Modulus
K	Initial Permeability
LE	Linear Elastic (constitutive model)
LEI	Linear Elastic Isotropic (constitutive model)
M	Permeability strain-dependency coefficient

mm	millimetre
MRI	Magnetic Resonance Imaging
μm	micrometre
Na+	Sodium cation
NI	Nanoindentation
OA	Osteoarthritis
OARSI	Osteoarthritis Research Society International
OC	Osteochondral
OP	Osteoporosis
P	Plastic (constitutive model)
pBV/TV	Plate Bone Volume Fraction
PG	Proteoglycan
pQCT	Peripheral Quantitative Computed Tomography
PRISMA	Preferred Reporting Items for Systematic Reviews and Meta-Analyses
pTb.S	Average Plate Trabecular Surface
pTb.Th	Average Plate Trabecular Thickness
qCT	Quantitative Computed Tomography
rBV/TV	Rod Bone Volume Fraction
SB	Subchondral Bone
SMI	Structural Model Index
TB	Trabecular Bone
tBMD	Volumetric Bone Mineral Density contributed by transverse trabeculae
Tb.N	Trabecular Number
Tb.Sp	Trabecular Separation
Tb.Th	Trabecular Thickness
T2D	Typer-2 Diabetes
US	Ultrasonography
VE	Visco Elastic (constitutive model)
η	Viscosity coefficient
